# Cardiovascular Magnetic Resonance Imaging in Myocardial Disease

**DOI:** 10.7759/cureus.58688

**Published:** 2024-04-21

**Authors:** Oana-Andreea Popa, Mihaela Amzulescu, Claudia Bugeac, Luminita Tomescu, Iulian M Slavu, Valeriu Gheorghita, Rosu Andrei, Adrian Tulin

**Affiliations:** 1 Cardiology, Agrippa Ionescu Emergency Clinical Hospital, Bucharest, ROU; 2 Cardiology, Centre Hospitalier Universitaire (CHU) Saint Pierre, Bruxelles, BEL; 3 Radiology, Agrippa Ionescu Emergency Clinical Hospital, Bucharest, ROU; 4 Anatomy, Carol Davila University of Medicine and Pharmacy, Bucharest, ROU; 5 Infectious Disease, Agrippa Ionescu Emergency Clinical Hospital, Bucharest, ROU; 6 Clinic of General Surgery, Agrippa Ionescu Emergency Clinical Hospital, Bucharest, ROU

**Keywords:** late gadonlinium enhancement, cardiology, cardiomyopathies, myocardial disease, cardiovascular magnetic resonance imaging

## Abstract

Cardiovascular magnetic resonance (CMR) is the central non-invasive imaging investigation for the evaluation of myocardial disease. It is the well-established gold standard for measuring cardiac chamber volumes, systolic function, and left ventricular mass, and it brings unique information for therapeutic decisions. In addition, its tissue characterization capability, through T1, T2, and T2* mapping, as well as early and late gadolinium enhancement (LGE) sequences, allows to differentiate in many cases among ischemic, inflammatory, and infiltrative heart disease and permits the quantification of myocardial fibrosis, providing valuable diagnostic and prognostic information. This review aims to highlight the main CMR features of different cardiomyopathies.

## Introduction and background

Cardiomyopathies (CMP) can be classified into two major categories: non-ischemic and ischemic [1]. Cardiovascular Magnetic Resonance (CMR) imaging not only provides accurate data about cardiac function, but its tissue characterization capabilities can discriminate between the two etiologies. CMR can detect myocardial edema, fibrosis, and extracellular infiltration with amyloid or intracellular storage of iron, glycogen, fat, etc., data that correlates with the results of histopathological examinations. This investigation provides essential information in establishing the etiology and prognosis of the underlying heart disease [2]. CMR is a complex investigation that requires highly trained staff and expensive equipment. Due to these limiting aspects, the rate of worldwide use cannot be evaluated. Non-ischemic cardiomyopathies are classified, according to the last ESC guidelines, in different specific phenotypes like dilated cardiomyopathy (DCM), non-dilated left ventricular cardiomyopathy (NDLVC), hypertrophic cardiomyopathy (HCM,) restrictive cardiomyopathy (RCM), arrhythmogenic right ventricular cardiomyopathy (ARVC) and non-differentiated cardiomyopathy like left ventricular non-compaction (NCLV) and Takotsubo Syndrome (TS) [3]. There are frequent overlaps between phenotypes, for example, cardiac sarcoidosis can express either as a DCM or RCM phenotype and sometimes can mimic ARCV; in this case, CMR is a key investigation and, along with the other imagistic tools, histopathological exams, and laboratory investigations, including the genetic tests, guides the final diagnosis. Serial follow-up CMR can evaluate disease progression and the response to the therapy (e.g., evaluation of iron deposition in haemochromatosis or late gadolinium enhancement (LGE) in myocarditis) and should be considered in all patients with cardiomyopathies. The aim of the review was to evaluate the utility and indications of CMR in different myocardial diseases. by analyzing and verifying the most recent publications. We used our own database of CMR images to depict what different cardiomyopathies resemble.

## Review

Literature search

We have conducted a computerized search of Medline (Pubmed), Science Direct, Cumulative Index to Nursing and Allied Health Literature (CINAHL), Cochrane database, and Google Scholar was performed to investigate the role of CMR in myocardial disease. As an example of a literature search, the following search terms: CMR and myocardial disease, CMR and cardiomyopathies, heart failure and CMR, CMR and dilated cardiomyopathy, CMR and myocarditis, CMR and hypertrophic cardiomyopathy, CMR and Anderson-Fabry disease, CMR and amyloidosis, CMR and sarcoidosis, CMR and glycogen storage disease, CMR and cardiac siderosis, CMR and hypereosinophilic cardiomyopathy, CMR and neuromuscular disorders, CMR and TakoTsubo cardiomyopathy, CMR and arrhythmogenic right ventricular cardiomyopathy CMR and non-compaction cardiomyopathy and CMR and ischemic cardiomyopathies. Abstracts were screened first, then a full-text assessment of the articles was performed, and finally, a narrative review was made with illustrative cases from our experience.

Non-ischemic cardiomyopathies

Dilated Cardiomyopathy (DCM)

**Figure 1:**
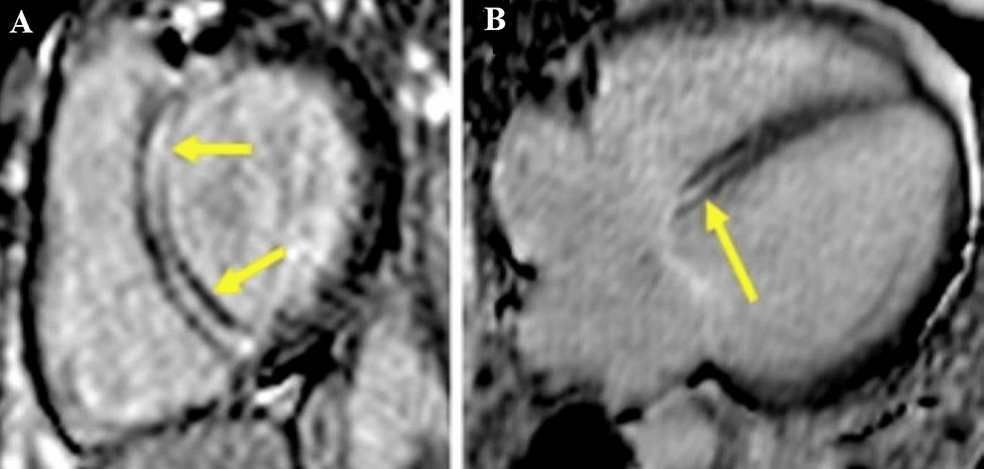
CMR images of a 45-year-old male patient diagnosed with dilated cardiomyopathy CMR: Cardiac magnetic resonance Late Gadolinium enhancement images (LGE) in short axis (A) and four-chamber views (B), respectively, showing mid-myocardial focal interventricular septal fibrosis (yellow arrowheads), suggesting an idiopathic cardiomyopathy. Image courtesy: Personal archive of Dr. Oana Popa. Patient consent was obtained.

Dilated cardiomyopathy represents the cardiomyopathy characterized by dilatation of the left ventricle (LV) and systolic dysfunction, alone or in association with dilatation or dysfunction of the right ventricle (RV), in the absence of abnormal loading conditions like volume or pressure overload or the presence of significant coronary atherosclerosis that can explain the occurrence of the global systolic dysfunction; in adults, it is defined as an LV end LV end-diastolic diameter >58 mm in males and >52 mm in females and an LV end-diastolic (LVEDV) index of ≥75 mL/m^2^ in males and ≥62 mL/m^2^ in females, in patient with a left ventricular ejection fraction (LVEF) less than 50% [1,3]. It is genetically determined in 30-40% of cases; in the rest of the cases, various causative factors are involved, such as infectious (viral, bacterial, fungal, or parasitic myocarditis), autoimmune, toxic (alcohol, cocaine, steroids), nutritional deficiency (thiamine, carnitine, selenium), endocrine disorders (diabetes mellitus, hypothyroidism, and hyperthyroidism) [3-4].CMR is currently routinely recommended to all patients with DCM, at least once in their lifetime, for etiological and prognostic information, being the gold standard in the evaluation of bi-ventricular function [5-6]. The focal myocardial fibrosis pattern, observed in 30-40% of DCM cases, is different from the subendocardial or the transmural pattern seen in ischemic heart disease and is often located at the level of the mid-myocardium of the interventricular septum (Figure1) or subepicardial in those with an inflammatory component [7]. It has been demonstrated that mid-myocardial fibrosis identified by CMR represents a negative prognostic factor, being associated with a higher risk of mortality from any cause, increased hospitalizations, and malignant arrhythmias (sustained ventricular tachycardia (VT)/non-sustained ventricular tachycardia (NSVT)[8].

Native T1 and extracellular volume (ECV) calculation can identify another type of fibrosis, namely diffuse interstitial myocardial fibrosis, which is due to reactive accumulation of collagen in the interstitial space [9], as opposed to the above-mentioned focal replacement myocardial fibrosis. It has been shown to be an independent negative prognostic factor, which correlates with ventricular volumes, contractility, and diastolic dysfunction [10-12]. CMR examination indications in DCM: Differential diagnosis between ischemic vs non-ischemic etiology [13-14]. Identification of specific etiologies (sarcoidosis, hemochromatosis etc). Before implantation of cardioverter defibrillator (ICD)/cardiac resynchronization therapy (CRT) [15]. Detection of intracardiac thrombi. Evaluation of global biventricular function, pre-treatment, and during follow-up, prognostic factor assessment of focal and interstitial myocardial fibrosis (Figure 1) [16-18].

Non-dilated left ventricular cardiomyopathy phenotypes (NDLVC) is a new subtype of DCM, described by the new ESC guidelines from 2023, which is characterized by the absence of LV dilatation despite global LV systolic dysfunction (an LVEF < 50%) with or without non-ischemic scar on LGE-CMR, or normal LV systolic function but with myocardial non-ischemic scar described by the LGE-CMR images. CMR is essential for the diagnosis and evaluation of this new phenotype [3].

Acute Myocarditis

**Figure 2:**
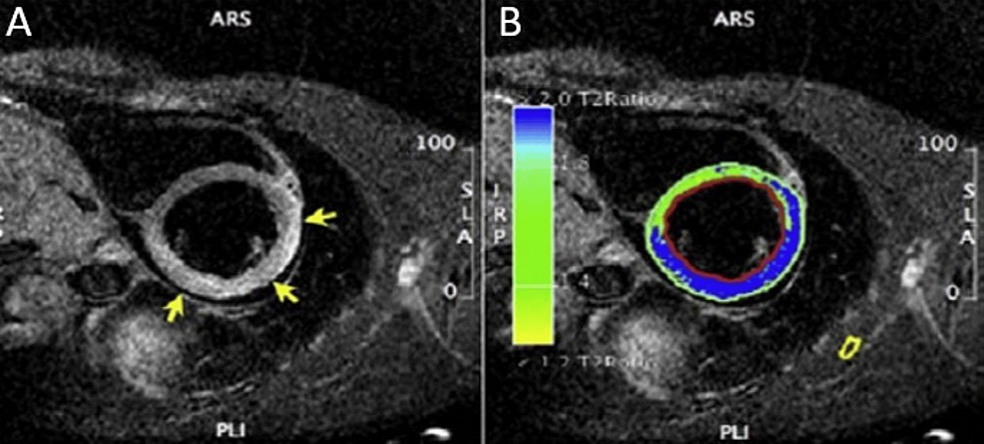
CMR examination of a 23-year-old male patient diagnosed with myocarditis-T2 images CMR: Cardiac magnetic resonance (A): T2-weighted CMR image showing subepicardial oedema in the inferolateral, inferior, and anterior segments. B: Computer-aided signal intensity analysis of the T2-weighted image with color-coded display of relative signal intensity, normalized to skeletal muscle. Blue indicates a signal intensity ratio of myocardium/skeletal muscle of ≥2.0, indicating oedema, and green indicates normal signal intensity. Image courtesy: Personal archive of Dr. Oana Popa. Patient consent was obtained.

**Figure 3:**
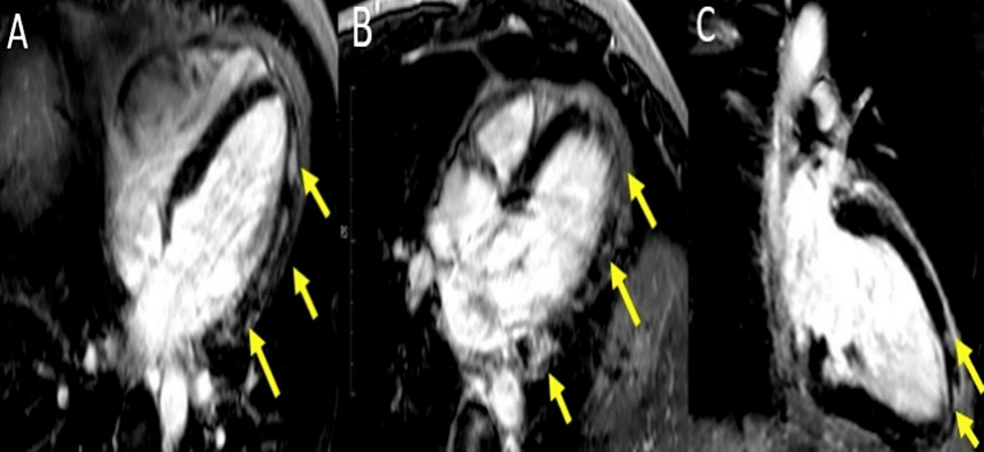
CMR examination of a 23-year-old male patient diagnosed with myocarditis-LGE images CMR: Cardiac magnetic resonance, LGE: Late Gadolinium Enhancement LGE acquired in longitudinal four-chamber (A), three-chamber (B), and two-chamber views (C), respectively, showing areas of sub-epicardial to mid-myocardial hyperenhancement (yellow arrowheads), not corresponding to a coronary artery distribution,  predominantly in inferolateral and anterolateral walls, suggestive of myocarditis. Image courtesy: Personal archive of Dr. Oana Popa. Patient consent was obtained.

It is defined as the inflammation of the heart muscle, which may lead to heart failure [1]. Even if the definite diagnosis can be confirmed by myocardial biopsy and histopathological examination, in recent years, CMR has become an important, non-invasive tool in its diagnosis by highlighting edema on T2 and T2 mapping images, hyperemia in early gadolinium enhancement (EGE) images, as well as identifying local fibrosis/necrosis in LGE images (Figures 2, 3) [19-20].

The 2009 Lake Louise diagnostic criteria for myocarditis were subsequently revised in 2018, thus requiring at least one major criterion for a positive diagnosis [21] (See Table 1).

**Table 1:** Revised Lake Louise Criteria LV: Left ventricle, ECV: Extracellular volume Reference: [21]

Main Criteria	Myocardial edema		
	Regional or global increase of native T2 mapping	Regional or global increase of T2 signal intensity –T2w images (as shown in Figure 2)	
	Non-ischemic myocardial injury		
	Regional or global increase of native T1	Regional or global increase of ECV	Regional non-ischemic LGE (Figure 3)
Supportive Criteria	Pericardial effusion		
	Systolic LV dysfunction: regional and global wall motion abnormality		

The presence of LGE correlates with areas of edema on T2 images and is associated with an unfavorable prognosis [22-23]. To evaluate the response to the treatment as well as the prognosis, it is recommended to recheck patient by CMR after three to six months [24-25].

Hypertrophic Cardiomyopathy (HCM)

**Figure 4:**
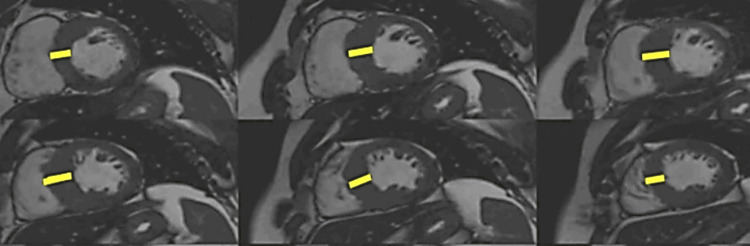
CMR examination of a 32-year-old female patient with hypertrophic cardiomyopathy-cine images CMR: Cardiac magnetic resonance Diastolic phase of a balanced steady-state free precession (B-SSFP) cine images, in short axis views, demonstrating asymmetrical septal hypertrophy, predominantly to the interventricular septum (yellow lines). Image courtesy: Personal archive of Dr. Oana Popa. Patient consent was obtained.

HCM is a primary myocardial condition characterized by the presence of left ventricular hypertrophy in the absence of a pressure overload condition that could explain it; In an adult patient, HCM is diagnosed by an LV wall dimension greater than 15 mm in any myocardial segment or more than 13 mm thickness in patients with relatives HCM diagnosed [3,26]. CMR is an essential tool for the accurate measurement of biventricular wall thickness (Figure 4), LV mass, and biventricular function and description of the hypertrophy pattern: symmetrical, asymmetrical, apical, as well as the evaluation of associated anomalies such as myocardial crypts, papillary muscle hypertrophy or abnormal insertion, systolic anterior motion of the mitral valve, the presence of intraventricular flow acceleration [27].

**Figure 5:**
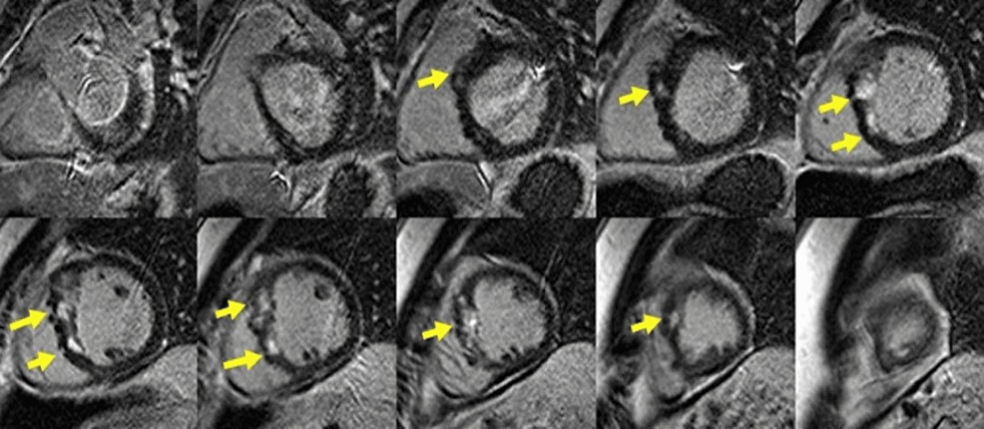
CMR examination of a 32-year-old female patient known with hypertrophic cardiomyopathy-LGE images CMR: Cardiac magnetic resonance, LGE: Late Gadolinium enhancement LGE acquired in short-axis views shows mid-myocardial LGE in an area of maximal hypertrophy (yellow arrows). Image courtesy: Personal archive of Dr. Oana Popa. Patient consent was obtained.

The information obtained is essential for the surgeon when considering mitral valve repair and/or septal myomectomy [28,29]. In many cases, CMR can differentiate among left ventricular hypertrophy phenocopies (sarcomeric vs non-sarcomeric like amyloidosis, Fabry, sarcoidosis) according to its distribution, fibrosis pattern, native T1 values, ​​and ECV measurement [30-32]. Myocardial fibrosis is found in a proportion of 33-86% [33,34] in patients with HCM; it is usually found at the mid-wall septal level, anterior wall, and usually in the areas where the wall is the thickest (Figure 5).

Another form of focal fibrosis found in HCM is at the insertion points of the septum to the RV level but is not pathognomonic. A fibrosis percentage of 15% of the LV mass quantified on LGE images is associated with an increased risk of sudden cardiac death (SCD) and early ICD implantation may be considered for the primary prevention of SCD [3,35-39]. ECV is most often increased in areas with hypertrophy and can differentiate the form of hypertrophy, in the intracellular forms the ECV is low or normal [40,41].

Anderson-Fabry Disease (AFD)

**Figure 6:**
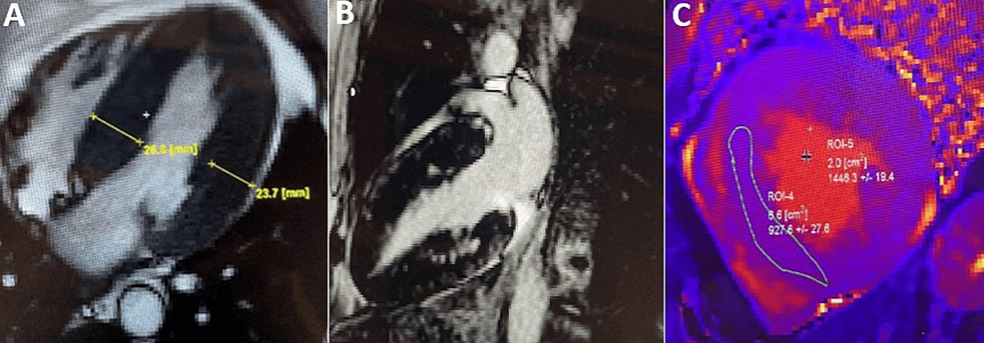
CMR examination of a 54-year-old male patient diagnosed after a dosage of alpha-galactosidase enzyme with Aderson-Fabry disease CMR: Cardiac magnetic resonance (A) Contrast-enhanced cardiovascular magnetic resonance diastolic frame of cine image in four-chamber view, showing severe left ventricular (LV) wall thickness predominantly to the interventricular septum of a maximum 27 mm (yellow line).  (B) Late gadolinium enhancement image in two chamber view shows an area of hyperenhancement in the basal mid myocardial inferior wall. (C) Native T1 mapping shows low T1 times (927 ms) suggesting a possible intracellular storage disease of the heart. Image courtesy: Personal archive of Dr. Oana Popa. Patient consent was obtained.

AFD is a systemic, X-linked disease, in which the alpha-galactosidase enzyme deficiency results in the progressive storage of intracellular glycosphingolipids [42]. Often, during the echocardiographic evaluation, this disease can mimic HCM. CMR highlights concentric, symmetrical, moderate to severe hypertrophy and, in advanced cases, focal mid-myocardial fibrosis at the level of the basal inferolateral wall. A particular pattern in it presents the native T1, which is low or “pseudonormal”, as well as the ECV value, which is normal, because the hypertrophy is due to the increase in the intracellular myocardial volume and not extracellular as in HCM [3,43] (Figure 6).

The T2 relaxation time in patients with AFD can be higher compared with that in HCM patients [44].

Cardiac Amyloidosis

**Figure 7:**
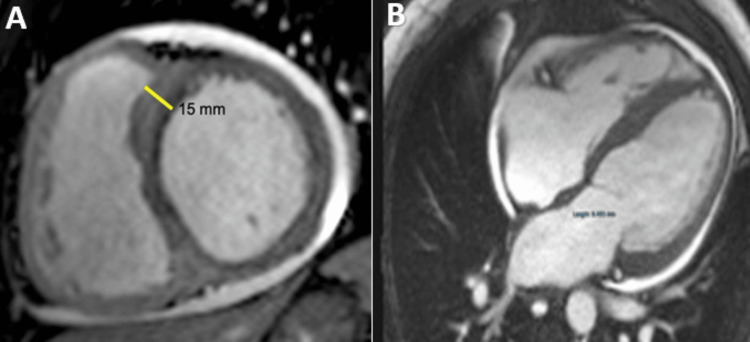
CMR examination of a 74-year-old female diagnosed with AL amyloidosis after fat pad biopsy-cine images CMR: Cardiac magnetic resonance, AL: Amyloid light-chain Diastolic phase of a balanced steady-state free precession (bSSFP) cine sequence of the short axis( A) and four chambers views (B), respectively.  The basal antero-septum is 15 mm thick (yellow line), while the other walls are not hypertrophied. Small, circumferential pericardial effusion is also noted. Image courtesy: Personal archive of Dr. Oana Popa. Patient consent was obtained.

It is a clinical disorder caused by the extracellular deposition of abnormal, insoluble amyloid fibers, which are stored in visceral organs. Diagnosis of cardiac amyloidosis can be invasive, for all types: demonstrating the presence of amyloid fibrils at endomyocardial biopsy (EMB) or revealing the amyloid deposits extracardiac in patients with suspected cardiac amyloidosis by echocardiography/CMR and non-invasive accepted only for the ATTR forms-the patients had positive single-photon emission computed tomography (SPECT) grade 2 or 3 myocardial radiotracer uptake and echocardiographic/CMR findings suggestive for cardiac amyloidosis [3,44-45]. In recent years, CMR has become the imaging method of choice for the diagnosis and evaluation of patients with cardiac amyloidosis [45]. Cardiac damage is manifested by restrictive hypertrophic or dilated cardiomyopathy secondary to pseudo-myocardial hypertrophy, often biventricular, “granular sparkling” aspect of the myocardium, with amyloid deposition also at the level of the interatrial septum and atrial walls [44-47] (Figure 7).

**Figure 8:**
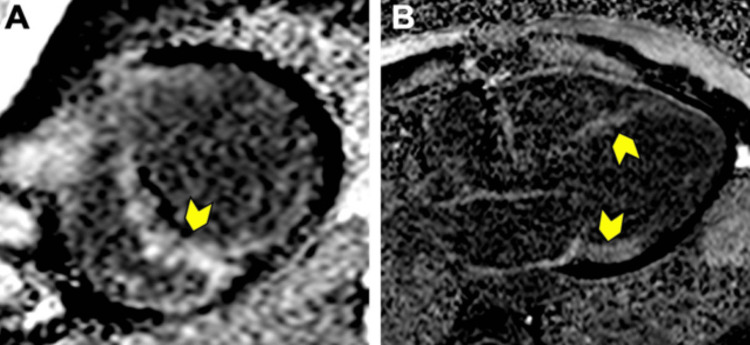
CMR examination of a 74-year-old female diagnosed with AL amyloidosis after fat pad biopsy-LGE image CMR: Cardiac magnetic resonance, LGE: Late Gadolinium Enhancement, AL: Amyloid light-chain LGE was acquired in short axis (A) and longitudinal three-chamber (B) views, respectively. Difficult nulling of the myocardium, with areas of transmural hyperenhancement (yellow arrowheads) without respecting a coronary artery distribution. Image courtesy: Personal archive of Dr. Oana Popa. Patient consent was obtained.

The particular aspect of diffuse subendocardial extensive fibrosis has a specificity of approximately 94% for amyloidosis (Figure 8).

LGE can also be highlighted in the RV and atrial walls. The binding of gadolinium to the amyloid proteins causes a faster washing of the contrast product from the blood pool, which appears blacker/darker than normal; this change is almost unique to amyloidosis [47-48]. In some patients with severe cardiac damage, transmural focal fibrosis can also be evident, being a sign of a poor prognosis [49]. EGE imaging can reveal intracavitary thrombi and areas of microvascular obstruction (MVO). The native T1 value, as well as ECV, are greatly increased in cardiac amyloidosis and correlate with the patient's prognosis. An ECV greater than 44% is an independent prognostic factor for mortality in AL amyloidosis [46,49-51].

Cardiac Sarcoidosis

**Figure 9:**
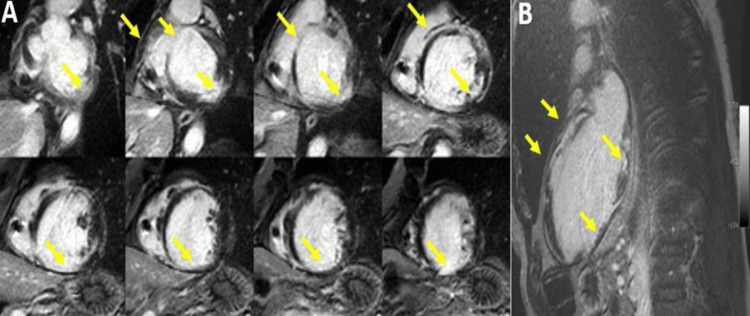
CMR examination of a 47-year-old man with a medical history of dilatated cardiomyopathy, normal coronary artery and permanent pacemaker for complete AV block-LGE images CMR: Cardiac magnetic resonance, LGE: Late Gadolinium Enhancement, AV: Atrioventricular, LV: Left ventricle, RV: Right ventricle LGE acquired in short axis (A) and longitudinal two-chamber (B) views, respectively, show extensive sub-epicardial to transmural patchy fibrosis at the level of LV and RV and raised the suspicion of cardiac sarcoidosis that was confirmed by endomyocardial biopsy (EMB). Image courtesy: Personal archive of Dr. Oana Popa. Patient consent was obtained.

Sarcoidosis is a systemic disease of unknown cause characterized by non-caseous granulomas that can affect almost any organ. The lung is most often affected and heart damage can be found in approximately 30% of cases [44,52-53]. CMR can highlight Global systolic dysfunction combined with bi-ventricular, regional, or global wall motion abnormalities, bi-ventricular dilatation, or hypertrophy. Slight increase in left ventricular mass secondary to granulomatous expansion. LGE images show areas of focal fibrosis, usually with mid-wall or sub-epicardial extension. Sometimes, they can even be transmural or sub-endocardial, but without a correlation to a coronary territory. The septum and lateral wall are often affected, but the papillary muscles, RV-free wall, and atria can also be involved. The localized increase of T2 in areas with granulomas can be highlighted in those with active disease and can be considered a marker for evaluating the response to treatment (Figure 9) [54-55]. 

The disease must be suspected in patients in the second to fifth decade with various degrees of A-V block or VT and sudden symptoms and signs of heart failure, but the definite diagnosis of CS is based on the histological examination by EMB [55]. Differential diagnosis can be made with other conditions that present extensive patchy LGE like arrhythmogenic cardiomyopathy (ARC), HCM, myocarditis, amyloidosis, DCM, Chagas disease or coronary artery disease. 18F-FDG-PET has been demonstrated to be the most accurate diagnostic imaging test for CS in patients with confirmed extra-cardiac histopathology for sarcoidosis [44,56].

Cardiac Siderosis

**Figure 10:**
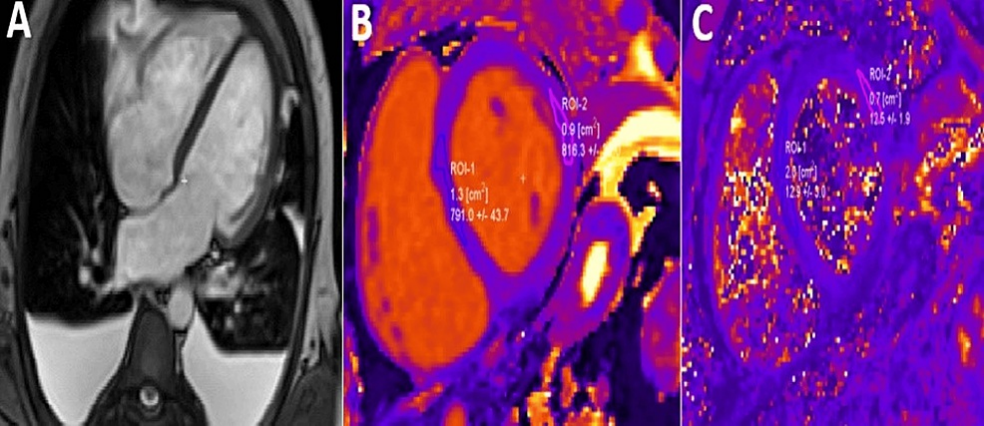
CMR exam of a 23-year-old female known with major beta thalassemia and frequent blood transfusions CMR: Cardiac magnetic resonance (A) Diastolic phase of a balanced steady-state free precession (BSSFP) cine sequence of 4 chambers view shows no dilated heart, with diastolic dysfunction. Bilateral pleural effusion is also noted, in patients with confirmed hemochromatosis. (B) Native T1 mapping shows low T1 times (791 ms). (C) Native T2* mapping shows a low value (12.9 ms); less than 20 ms (on a 1.5 T system) indicates significant iron deposition. Image courtesy: Personal archive of Dr. Oana Popa. Patient consent was obtained.

Cardiac siderosis is encountered in patients with hemochromatosis, a genetic disease characterized by a disorder of iron metabolism that causes an overload of iron and its deposition in parenchymal organs, or independent transfusion patients, like in major thalassaemia. It is a storage, not an infiltrative disease, the iron is stored in the sarcoplasm and not interstitium [44]. Cardiac damage depends on the site and the amount of iron stored in the heart, most often the ventricles are affected with an imaging phenotype of restrictive cardiomyopathy, sometimes even dilatative, but in the early stages affects just de diastolic LV function. CMR can quantify myocardial iron deposits using T2* sequences, being the only non-invasive method that can determine this, well-validated by histopathology. A myocardial T2* value of less than 20 ms (1.5 T system) indicates significant iron deposition in the myocardium. This parameter is used to initiate therapy with iron chelators and to monitor the response to it. Native T1 is decreased and can be a complementary tool to T2* in the diagnosis of cardiac iron overload [57-59] (Figure 10).

Glycogen Storage Disease (GSD)

**Figure 11:**
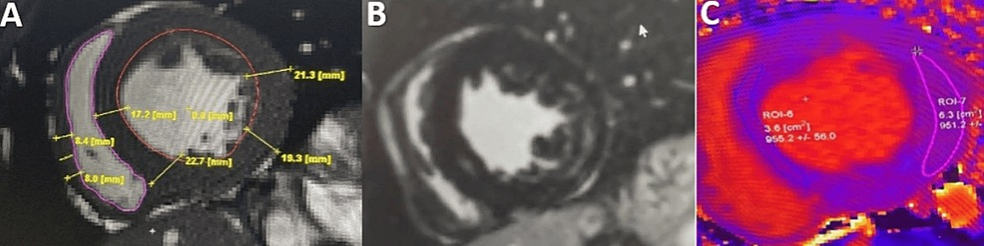
CMR examination of a 19-year-old male patient diagnosed after genetic tests with Pompe disease CMR: Cardiac magnetic resonance (A) Contrast enhanced cardiovascular magnetic resonance diastolic frame of cine image in short chamber view, showing severe left ventricular wall thickness and free right ventricular thickness. (B) Late gadolinium enhancement image in short axis shows an area of hyperenhancement in the mid myocardial inferolateral wall. (C) Native T1 mapping shows low T1 times (955 ms) while the calculation of the extracellular volume (ECV) showed no interstitial expansion (ECV- 18%) suggesting a possible intracellular storage disease of the heart. Image courtesy: Personal archive of Dr. Oana Popa. Patient consent was obtained.

Glycogenosis represents a group of metabolic disorders characterized by abnormalities of the enzymes that regulate the synthesis and degradation of glycogen. There are 11 different types of GSD and the most well-known are Pompe disease (GSD tip IIa), Danon disease (GSD tip IIb) and PRKAG2 syndrome [3,44]. Cardiac damage is manifested by severe left ventricular hypertrophy, which can mimic HCM, and in the advanced stages, chamber dilation and ventricular dysfunction may develop [44,60]. In Danon's Disease, CMR can highlight extensive sub-endocardial fibrosis, mainly at the level of the apical segments with sparing of the basal septum. In Pompe disease, the LGE is rare and can be seen in sub-endocardium of the lateral and anterior walls (Figure 11) [44,60].

Hypereosinophilic Cardiomyopathy

**Figure 12:**
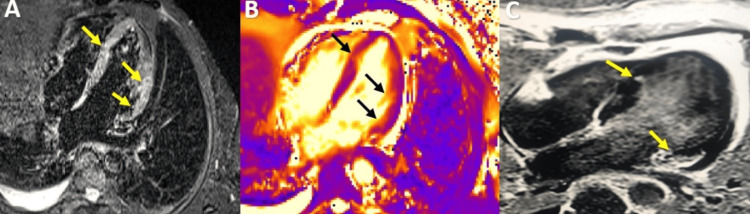
CMR examination of a 41-year-old male patient with toxocariasis with cardiac involvement CMR: Cardiac magnetic resonance Horizontal long axis (HLA) views: T2-weighted STIR image (A) and T2 mapping image (B) showing subendocardial oedema in the inferolateral wall and medium inferior septum (yellow arrows).  (C) Late gadolinium enhancement images show focal fibrosis at the level of basal inferolateral wall and medium inferior septum (yellow arrows).  Right pleural effusion is present. Image courtesy: Personal archive of Dr. Oana Popa. Patient consent was obtained.

Eosinophilia can be associated with endomyocardial inflammation that often leads to fibrosis and, ultimately, restrictive cardiomyopathy causing endomyocardial fibrosis (EMF). It occurs in several conditions, such as hypereosinophilic syndrome affecting the heart (former Loeffler endocarditis), tropical and non-tropical endomyocardial fibrosis, or eosinophilic granulomatosis with polyangiitis (former Churg-Strauss syndrome) [44]. It has three stages of evolution: the first is eosinophilic myocarditis (acute necrotic stage, Figure 12), the intermediate stage is characterized by inflammation of the endocardium around the apex with extension to the middle segments and papillary muscles and the formation of apical thrombi and the third phase is the fibrotic stage that reveals endocardial thickening and restrictive pattern with the apical obliteration of LV and/or RV [44,55,61].

CMR shows the apical thrombus, which can often be misdiagnosed as apical hypertrophy on echocardiography, the early Gadolinium enhancement images being the ones that make the diagnosis. LGE images can highlight circumferential subendocardial fibrosis at the level of the apical segments, sometimes also the middle segments and papillary muscles, and the T2 images show hypersignal at this level [62-63].

Cardiomyopathy Associated to Neuromuscular Disorders

**Figure 13:**
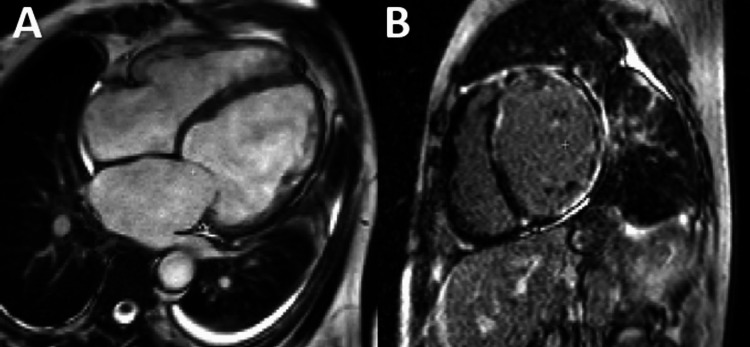
CMR examination of a 45-year-old male patient known with Duchenne neuromuscular dystrophy CMR: Cardiac magnetic resonance (A) Contrast enhanced cardiovascular magnetic resonance diastolic frame of cine image in four-chamber view, showing severe left ventricular (LV) dilatation and severe systolic dysfunction(B) Late gadolinium enhancement image in short axis shows extensive area of hyperenhancement, predominantly subendocardial and epicardial in all LV segment and  in the right ventricle free wall. Image courtesy: Personal archive of Dr. Oana Popa. Patient consent was obtained.

Neuromuscular dystrophies are primitive diseases of the skeletal and/or cardiac muscles, which over time cause muscle weakness and, from a cardiac point of view, dilated cardiomyopathy with systolic dysfunction, often caused by atrioventricular conduction disorders [59,64]. CMR shows an aspect of dilated cardiomyopathy with areas of focal fibrosis predominantly at the level of the lateral and inferolateral wall, with T2 hypersignal at this level in acute forms of the disease. LGE may be noted predominantly in the lateral and inferolateral walls, with a non-ischemic pattern (Figure 13) [59,64-65].

Left Ventricular Non-Compaction (LVNC)

**Figure 14:**
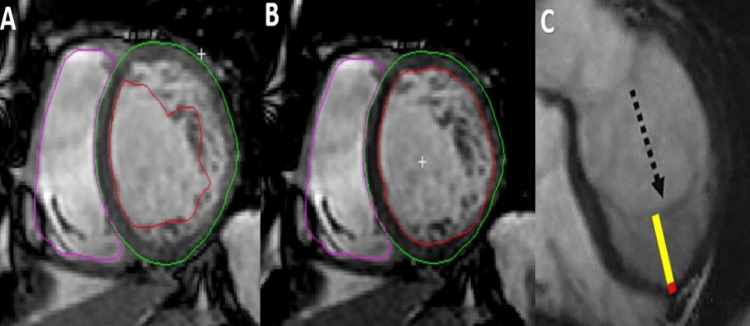
CMR examination of a 27-year-old male patient with dilatated cardiomyopathy suggestive for non-compaction CMR: Cardiac magnetic resonance (A-B) Contrast enhanced cardiovascular magnetic resonance diastolic frame of cine image in short chamber view, with a measure of the non-compacted myocardium (Jacquier criteria). (C)  Contrast enhanced cardiovascular magnetic resonance diastolic frame of cine image in a four-chamber view shows the presence of a noncompact (yellow line)/compact (red line) ratio of more than 2.3 (Petersen criteria). Image courtesy: Personal archive of Dr. Oana Popa. Patient consent was obtained.

Cardiomyopathy is characterized by extensive myocardial trabeculations in association with a thin and recessed epicardium that communicates with the cavity of the LV, where a thrombus can often form. Over time it can progress to ventricular systolic dysfunction and malignant arrhythmias [66]. CMR has become an important tool in the diagnosis of patients with left ventricular non-compaction (LVNC), several diagnostic criteria have been proposed over time, the most frequently used is the one proposed by Petersen which requires the presence of a noncompact/compact ratio of 2.3 [67] and Jacquier describes as a diagnostic criterion the presence of a trabeculated mass > 20% [68]. All these diagnostic criteria by CMR proved to be suggestive for LVNC but non-specific and too sensitive, therefore overestimating the disease which is why it is recommended to integrate clinical, preclinical, genetic data and family history in the diagnostic workup (Figure 14) [69].

Arrhythmogenic Right Ventricular Cardiomyopathy (ARVC)

It is a genetic disease characterized by predominantly fibrofatty infiltration of the right ventricle or both ventricles, it is associated with malignant rhythm disorders and SCD in young people [70]. In recent years, CMR has become a basic non-invasive imaging method in the diagnosis of ARVC patients. Several criteria have been defined over time, the “Padua Criteria” currently being used, these are grouped in six types: morpho-functional and structural ventricular abnormalities, depolarization and repolarization electrocardiographic alterations, ventricular arrhythmias, and familial/genetic history [70,71]. CMR can describe the morpho-functional and structural ventricular abnormalities like RV dilatation, RV dysfunction, presence of akinetic, dyskinetic areas, aneurysms at the RV level, often affecting the RV triangle: subtricuspid area, RV ejection tract, and RV apex and in some cases, it can describe LV lesions: systolic dysfunction, regional wall motion abnormality and/or presence of fibrosis [4] (see Table 2).

**Table 2:** CMR criteria for ARVD CMR: Cardiac magnetic resonance, ARVD: Arrhythmogenic right ventricular cardiomyopathy; EDV: End diastolic volume; EF: Ejection fraction; ITF: International Task Force; LGE: Late gadolinium enhancement; LV: Left ventricle; RV: Right ventricle. Reference [70].

	Right ventricle (upgraded 2010 ITF diagnostic criteria)	Left ventricle (new diagnostic criteria)
Morpho-functional ventricular abnormalities	Major •Regional RV akinesia, dyskinesia, or bulging plus one of the following: -global RV dilatation -global RV systolic dysfunction	*Minor* •Global LV systolic dysfunction with or without LV dilatation *Minor* •Regional LV hypokinesia or akinesia of LV free wall, septum, or both
Structural myocardial abnormalities	*Major* •Transmural LGE (stria pattern) of≥1 RV region(s) (inlet, outlet, and apex in 2 orthogonal views)	*Major* •LV LGE (stria pattern) of ≥1 Bull's Eye segment(s) (in 2 orthogonal views) of the free wall (subepicardial or midmyocardial), septum, or both (excluding septal junctional LGE)

**Figure 15:**
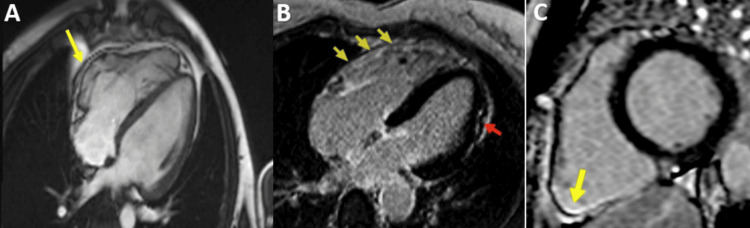
CMR exam of a 29-year-old male diagnosed with ARVC after positive genetic tests for desmoplakine mutation CMR: Cardiac magnetic resonance Contrast enhanced cardiovascular magnetic resonance diastolic frame of cine image in four-chamber view shows micro-aneurysms at the level of right ventricle (RV) free wall. Late gadolinium enhancement image (LGL) four-chamber (B) and short axis (C), respectively, showing fibrosis of the level of RV free wall (yellow arrows) and subendocardial fibrosis of the level of inferolateral wall (red arrow). Image courtesy: Personal archive of Dr. Oana Popa. Patient consent was obtained.

**Figure 16:**
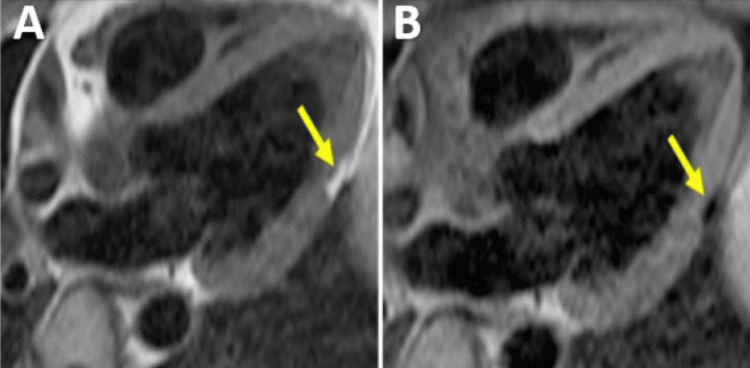
CMR exam of a 29-year-old male diagnosed with ARVC after positive genetic tests for desmoplakine mutation CMR: Cardiac magnetic resonance, ARVC: Arrhythmogenic right ventricular cardiomyopathy Horizontal long axis (HLA) views. Native T1 image (A) and T1 fat-saturation image (B) showing subepicardial fat in the inferolateral wall (yellow arrows). Image courtesy: Personal archive of Dr. Oana Popa. Patient consent was obtained.

T1 images may highlight fatty infiltration at the level of the RV free wall, sometimes this can also be evident at the LV level in those with biventricular damage (Figures 15, 16.), but with reduced sensitivity and specificity, and LGE images describe the presence of fibrosis in the areas corresponding to fatty infiltration, in those with LV damage, subepicardial fibrosis can be depicted, usually at the level of the lateral or inferolateral wall, sometimes also at the septal level or even subepicardial circumferential infiltration in those with predominantly LV damage [72-73].

Takotsubo Syndrome (TS)

**Figure 17:**
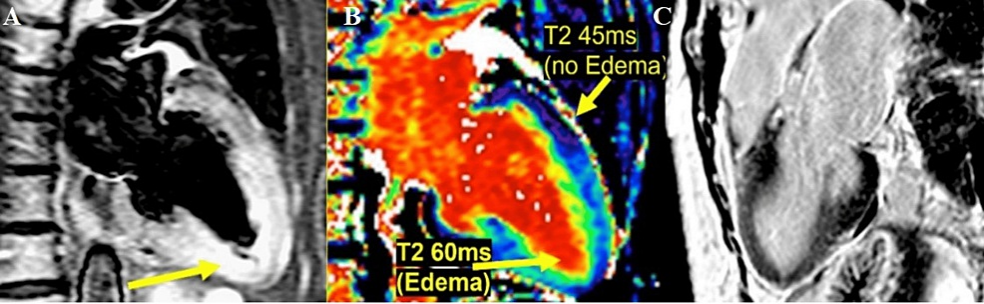
CMR examination of a 68-year-old female patient with Takotsubo cardiomyopathy CMR: Cardiac magnetic resonance Vertical long axis (VLA) views from left to right : A shows a T2-weighted STIR image. B shows T2 mapping image showing apical circumferential subepicardial oedema (yellow arrows). C shows late gadolinium enhancement image shows no area of hyperenhancements suggesting the absence of focal myocardial fibrosis. Image courtesy: Personal archive of Dr. Oana Popa. Patient consent was obtained.

It is a cardiomyopathy manifested by acute reversible ventricular systolic dysfunction, with symptoms like those of an acute coronary syndrome, but without significant coronary stenosis, triggered most of the time by strong stress, and, in approximately three to six months, recovery is noted complete [74-75]. The diagnosis is supported by extended kinetic disorder at the LV and/or RV level, that does not respect a vascular territory and is often circumferentially, typically the apical segments but the middle or basal segments can also be involved, with hypercontractility of the rest segments, absence of a culprit coronary lesion, new ECG changes over/under elevations of the ST segment, negative T waves, intraventricular conduction disorder, long QT, which resolves after the acute phase, increase in markers of myocardial necrosis: hsT and NT proBNP, complete recovery at three months of ventricular function [76-77]. CMR is an important imaging technique in the diagnosis of Takotsubo cardiomyopathy, highlighting global or regional kinetics disorder, accurate estimation of biventricular function, presence of complications such as obstruction in the LV ejection tract, quantification of valvulopathies, intracardiac thrombosis and the presence of pericardial effusion [78-79], myocardial edema evaluated by T2-weighted (T2-W) images, often the myocardial edema is circumferential, transmural, at the level of the apex with extension to the middle segments, corresponding to the areas with kinetic disorders, this is reversible and remits in three to six months [80-81], myocardial necrosis: the absence of LGE was described in most cases of TS, but some studies highlighted the presence of LGE in some patients with TS, the explanation for its appearance being given by the increase in myocardial interstitial volume and not by the occurrence of irreversible myocardial necrosis/fibrosis [78,82], myocardial perfusion is normal in the majority of cases, but sometimes this can highlight sub-endocardial perfusion defects in the apical and middle segments, due to severe microvascular dysfunction [82-83]. Follow-up at three-six months shows normalization of bi-ventricular function, reduction of ventricular volumes, disappearance of edema and absence of LGE. Therefore, CMR becomes the gold-standard for diagnosing CM TS and highlighting its complications, being able to differentiate it from classic acute coronary syndrome (Figure 17).

Ischemic Cardiomyopathy

CMR can accurately identify cardiac anatomy, bi-ventricular function, myocardial ischemia and/or viability and can differentiate between acute and chronic myocardial injuries with edema imaging [84]. Left ventricle ejection fraction (LVEF) is an important prognostic factor in the ischemic heart disease and its value is establishing the need of ICD and CRT therapy systems [85-86]. With the conventional T2-weighted images, CMR can detect myocardial edema and can differentiate between acute and chronic infarcts [10,87-88].

**Figure 18:**
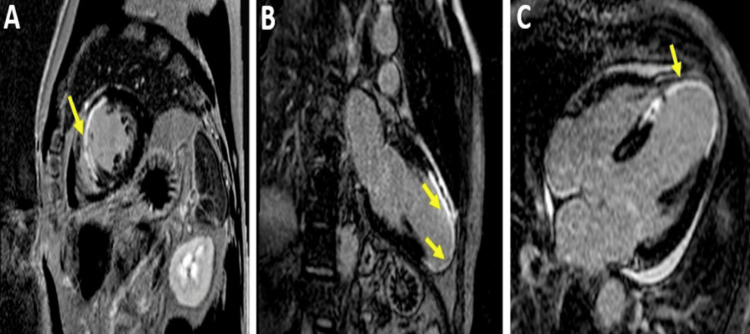
. CMR examination of a 67-year-old male patient with large anterior infarct CMR: Cardiac magnetic resonance Late gadolinium enhancement images short axis (A), longitudinal two-chamber (B) and three-chamber (C), respectively, showing transmural fibrosis (>75%) (yellow arrows)-non-viable territory. Note small circumferential pericardial effusion. Image courtesy: Personal archive of Dr. Oana Popa. Patient consent was obtained.

Using contrast CMR are obtaining LGE images that can differentiate between ischemic and non-ischemic etiology, in ischemic heart disease they are having typical subendocardial to transmural aspect, respecting a coronary artery distribution [89]. The LGE pattern can assess myocardial viability and likelihood for recovery after revascularization [90]. A transmurality less than < 50% has been shown to predict a very high likelihood for functional recovery, while functional improvement in segments with scar transmurality of > 50% was only 8%, and in a scar > than 75% the viability is null (Figure 18) [59].

Stress CMR

**Figure 19:**
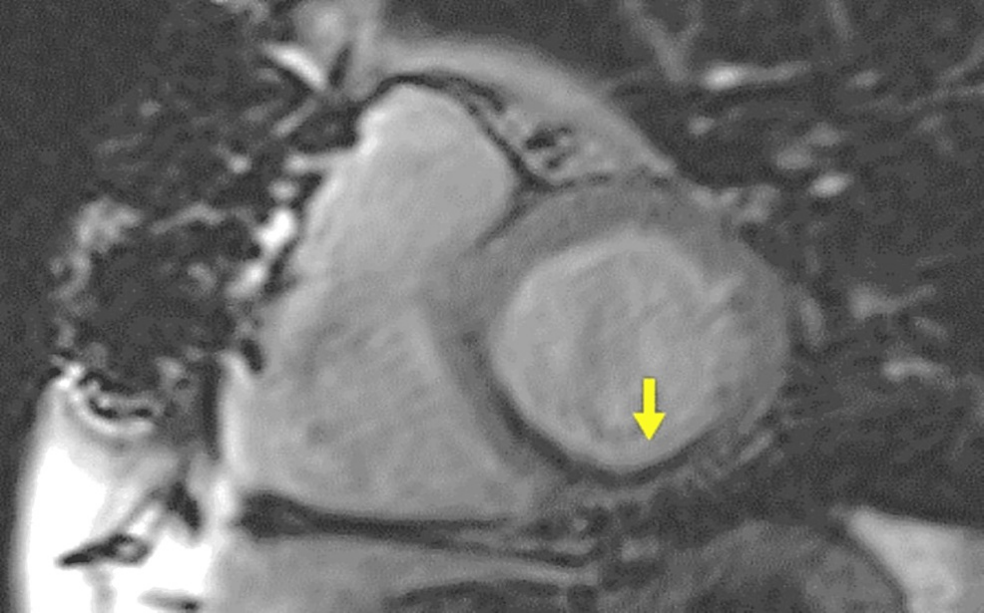
Stress CMR with adenosine in a patient known with triple coronary artery by-pass and heart failure symptoms CMR: Cardiac magnetic resonance Perfusion short axis view, shows perfusion defect in the inferior wall (yellow arrow). Image courtesy: Personal archive of Dr. Oana Popa. Patient consent was obtained.

It is performed with pharmacological stress agents that are two types: vasodilators like adenosine or regandenson and positive inotropes and chronotropes such as dobutamine. Vasodilators are increasing blood flow in non-stenotic coronary arteries but not in severely stenotic arteries. During maximal stress, a contrast-enhanced perfusion acquisition is performed, and a typical perfusion defect is visualized as an endocardial to transmural dark zone while the rest of the myocardium is enhanced by the contrast agent (Figure 18). Dobutamine is administered to increase myocardial oxygen demand comparable to physical exercising [91]. It can be given at a low dose to recruit the contractile reserve of hibernating myocardium or a high dose to induce myocardial ischemia in territories with significant coronary stenosis that can be depicted by the onset of wall-motion abnormalities. High dose dobutamine CMR is superior to dobutamine stress echo regarding diagnostic accuracy [92]. It has been demonstrated that the presence of inducible ischemia or an ejection fraction of < 40% independently predicts future myocardial infarction or cardiac death (Figure 19) [93].

## Conclusions

In the last decade, CMR has been included in the routine clinical protocols for the evaluation of heart diseases in different ranges of cardiac pathologies. With its unique ability to characterise the composition and perfusion of the myocardium, CMR can different between ischemic and non-ischemic etiology for cardiomyopathy and also to establish the etiology of non-ischemic pathologies by using multiple different sequences such as LGE, T1 mapping with ECV measurements, T2 mapping, and T2*, that allows visualization (and in some cases quantification) of tissue alterations such as iron deposits, edema, fibrosis, etc., with great diagnostic accuracy.

Several recent studies have shown the value of CMR for risk assessment for certain types of cardiomyopathies (e.g., sudden cardiac death and VT in hypertrophic cardiomyopathy and dilated cardiomyopathy), CMR has been increasingly used to ensure the best treatment for patients at the right time. In conclusion, CMR is one of the most important imaging tools in the evaluation of cardiomyopathies. This review demonstrated its power in diagnosing, prognosis, and developing treatment strategies across the large spectrum of heart disease. It should be performed in all patients with myocardial disease involvement, but this depends on the local availability and expertise.
